# Comprehensive pan-cancer analysis and experiments revealed R3HDM1 as a novel predictive biomarker for prognosis and immune therapy response

**DOI:** 10.3389/fgene.2024.1404348

**Published:** 2024-09-23

**Authors:** Jiawei Liu, Zhitong Bing, Junling Wang

**Affiliations:** ^1^ School of Public Health, Lanzhou University, Lanzhou, Gansu, China; ^2^ Institute of Modern Physics, Chinese Academy of Sciences, Lanzhou, Gansu, China; ^3^ Gansu Laboratory of Isotope, Gansu Provincial Laboratory, Lanzhou, Gansu, China

**Keywords:** R3HDM1, diagnosis, pan-cancer, prognosis, TME, LUAD

## Abstract

**Background:**

R3HDM1, an RNA binding protein with one R3H domain, remains uncharacterized in terms of its association with tumor progression, malignant cell regulation, and the tumor immune microenvironment. This paper aims to fill this gap by analyzing the potential of R3HDM1 in diagnosis, prognosis, chemotherapy, and immune function across various cancers.

**Methods:**

Data was collected from the Firehost database (http://gdac.broadinstitute.org) to obtain the TCGA pan-cancer queue containing tumor and normal samples. Additional data on miRNA, TCPA, mutations, and clinical information were gathered from the UCSC Xena database (https://xenabrowser.net/datapages/). The mutation frequency and locus of R3HDM1 in the TCGA database were examined using the cBioPortal. External validation through GEO data was conducted to assess the differential expression of R3HDM1 in different cancers. Protein expression levels were evaluated using the Clinical Proteomics Tumor Analysis Alliance (CPTAC). The differential expression of R3HDM1 was verified in lung adenocarcinoma cell lines and normal lung glandular epithelial cells via RT-qPCR. Cell migration and proliferation experiments were conducted by knocking down the expression of R3HDM1 in two lung adenocarcinoma cell lines using small interfering RNA. The biological role of R3HDM1 in pan-cancer was explored using the GSEA method. Multiple immune infiltration algorithms from the TIMER2.0 database was employed to investigate the correlation between R3HDM1 expression and the tumor immune microenvironment. Validation of transcriptome immune infiltration was based on 140 single-cell datasets from the TISCH database. The study also characterized a pan-cancer survival profile and analyzed the differential expression of R3HDM1 in different molecular subtypes. The relationship between R3HDM1 and drug resistance was investigated using four chemotherapy data sources: CellMiner, GDSC, CTRP and PRISM. The impact of chemicals on the expression of R3HDM1 was explored through the CTD database.

**Result:**

The study revealed differential expression of R3HDM1 in various tumors, indicating its potential as an early diagnostic marker. Changes in somatic copy number (SCNA) and DNA methylation were identified as factors contributing to abnormal expression levels. Additionally, the study found that R3HDM1 expression is associated with clinical features, metabolic pathways, and important pathways related to metastasis and the immune system. High expression of R3HDM1 was linked to poor prognosis across different tumors and altered drug sensitivity. Furthermore, the expression of R3HDM1 showed significant correlations with immune modulatory molecules and biomarkers of lymphocyte subpopulation infiltration. Finally, the study highlighted four chemicals that could influence the expression of R3HDM1.

**Conclusion:**

Overall, this study proposes that R3HDM1 expression is a promising biomarker for predicting the prognosis of cancer, especially lung adenocarcinoma, and the efficacy of immunotherapy, demonstrating the rationale for further exploration in the development of anti-tumor therapies.

## 1 Introduction

Cancer encompasses a complex array of diseases that currently pose a significant obstacle to global life expectancy. It primarily arises from the progressive development of uncontrolled cell growth and evasion of normal cell death, eventually spreading to adjacent organs and tissues over time. According to the 2020 statistics from the World Health Organization (WHO), cancer causes approximately ten million deaths worldwide, with the number of cancer patients remaining high and continuing to rise (Nassar and Blanpain, 2016). Given that cancer cells stem from normal cells, conventional drug treatments not only target cancer cells but also exhibit significant toxicity to non-cancerous cells in the body. Therefore, there is an urgent need for novel cancer targeted therapies that offer increased efficacy and reduced side effects (Phillips et al., 2006; Hanahan and Weinberg, 2011).

R3HDM1, also known as an RNA-binding protein with an R3H domain, has emerged as a pivotal focus in cancer research (Fukushi et al., 2021). Despite being an uncharacterized protein, the role of R3HDM1 in tumor progression, malignant cell regulation, and the tumor immune microenvironment remains largely unexplored. Understanding the significance of R3HDM1 in the context of cancer could potentially offer new insights into its diagnostic, prognostic, and immunotherapeutic value across a spectrum of cancer types. This study aims to comprehensively investigate the functional implications of R3HDM1 in cancer development and progression. By collecting and analyzing data from various databases and conducting vitro cellular experiments, this research seeks to elucidate the association between R3HDM1 expression levels in tumors and their clinical features, metabolic pathways, and immune modulation. The findings from this study are expected to shed light on how elevated R3HDM1 expression correlates with poor prognosis and altered drug sensitivity in different cancer types. Furthermore, exploring the relationship between R3HDM1 expression and immune modulatory molecules as well as lymphocyte subpopulation infiltration biomarkers could provide valuable insights into the role of R3HDM1 in the tumor immune microenvironment. *In vitro* cellular experiments were conducted to validate the differential expression of R3HDM1 between lung adenocarcinoma cell lines and normal lung glandular epithelial cells. Overall, this investigation aims to establish R3HDM1 as a promising molecular marker for predicting cancer patient outcomes and assessing the efficacy of immunotherapeutic interventions, thereby contributing to the development of more effective cancer treatments and potentially novel anti-tumor therapies.

## 2 Materials and methods

### 2.1 R3HDM1 multidisciplinary expression analysis in pan-cancer

First, we detected the differences in R3HDM1 between tumor and normal tissues in TCGA data. Then, we examined the differences in R3HDM1 expression between tumor and normal tissues in paired samples from TCGA cancer subgroups. Finally, we expanded the sample size of normal tissues by combining the data from TCGA and GTEx, and conducted differential analysis using the Wilcoxon test (*P < 0.05; ** *P* < 0.01; *** *P* < 0.001; **** *P* < 0.0001). The gene expression distribution in various organs was visualized using the gganatogram package. We used the pROC package to assess the potential role and significance of R3HDM1 in pan-cancer diagnosis and validated protein expression in external gene transcriptome levels in GEO database and CPTAC data. Logistic regression was performed to further validate the accuracy of the Wilcoxon test in four cohorts. It is worth noting that we conducted RT-qPCR experiments to verify the differential expression of R3HDM1 in normal lung epithelial cell line BEAS-2B and lung adenocarcinoma cell lines A549 and H1299.

### 2.2 Somatic copy-number alteration (SCNA), mutation analysis and DNA methylation analysis

We use the cBioPortal website for mutation-related analysis and visualization, including the frequency of changes, mutation types, CNA data, and gene mutation locations for all cancers (Cerami et al., 2012). Somatic cell copy number alterations (SCNA) and mutation can enhance the copy number changes of R3HDM1, with over 5% considered high-frequency SCNA. We calculate the Spearman correlation between the expression value and the copy number segment value of R3HDM1 to evaluate the association between SCNA and R3HDM1 expression. We use the Bioconductor R package “IlluminaHumanMmethylation-450kanno.ilmn12. hg19” to annotate methylation probes for R3HDM1 promoter. We test the differential methylation of R3HDM1 in tumor and normal samples using the Wilcoxon rank sum test and use a significance cutoff value of 0.05 to identify genes with significantly low or high methylation. We calculate the Spearman correlation between gene transcription expression and promoter DNA methylation Beta values, considering it significant if the *P*-value is < 0.05 (Mei et al., 2017; Zheng et al., 2019). Additionally, we calculate the Spearman correlation between R3HDM1 and 10 genomic feature scores.

### 2.3 Analysis of immune cell infiltration and assessment of the anti-cancer immune response

Utilizing TCGA tumor data, we employed seven different methods in TIMER2.0 to estimate the relationship between R3HDM1 expression levels and infiltration levels in various cells within the tumor immune microenvironment (Li T. et al., 2020). To investigate the cell types expressing R3HDM1 in tumor tissues, we performed in-depth single-cell analysis on 140 datasets from the TISCH database and visualized the findings (Han et al., 2023). Additionally, UMAP plots displaying the expression patterns of R3HDM1 in NSCLC_GSE143423 and NSCLC_GSE148071 were obtained from the TISCH database. Subsequently, visualizations were conducted to explore the relationship between R3HDM1 expression in different cells in NSCLC_GSE143423 and NSCLC_GSE148071 and functional pathways. Analysis on the impact of R3HDM1 expression levels on anti-cancer immune status in 32 cancer types was then carried out using the Tumor Immune Phenotype (TIP) database (http://biocc.hrbmu.edu.cn/TIP), with scoring performed for each step (Xu et al., 2018). Furthermore, the differences in immune activity scores between the high and low R3HDM1 expression groups were calculated. Visualization of the results was achieved using the R package pHeatmap (v1.0.12).

### 2.4 Prediction of immunotherapy and immune checkpoint inhibitor responses

We used the EaSIeR model and MeTIL scores to forecast the responses to anti-tumor immune checkpoint inhibitors based on RNA-seq data. EaSIeR is a predictive tool for biomarker-guided immunotherapy, relying on a model rooted in cancer-specific immune responses (Lapuente-Santana et al., 2021). MeTIL scores indicate the infiltration levels of T cells, NK cells, B cells, Tregs, and cytotoxic T lymphocyte (CTL) function (Jeschke et al., 2017).

### 2.5 Analysis of pathways and functional mechanisms of R3HDM1

We calculated the differential genes between the high-expression group and the low-expression group of R3HDM1 in pan-cancer. If a gene shows differential expression in more than five tumors, we defined it as an R3HDM1-related gene and used the clusterProfiler package for KEGG pathway enrichment analysis to identify the conservative function or biological pathways participated by this gene in pan-cancer (Wu et al., 2021). Research on complex diseases such as cancer has shown that the activity levels of proteins, such as expression and modification, have a significant impact on the occurrence and development of diseases. Changes in protein levels and structures have been proven to play a key role in tumor development but are not reflected in genetic changes. We used Spearman correlation analysis to identify the correlation between R3HDM1 expression and protein content identified by the RPPA method from the TCPA database (Li et al., 2017). The Sankey diagram visualized the results with correlation coefficients greater than 0.4 in all tumors. CancerSEA redefined 14 functional states (Yuan et al., 2019). In addition, we collected 14 classic tumor-related pathways from the KEGG database. We used the z-score parameter in the GSVA R package to calculate the gene sets of the 14 functional states and conducted Pearson correlation analysis (Lee et al., 2008). To determine the pathways associated with genes, we divided the samples of each tumor type into two groups, including the top 30% and the bottom 30%, and conducted gene set enrichment analysis (GSEA) to explore the gene set activation or inhibition in the high-expression group compared to the low-expression group in different tumors (Subramanian et al., 2005). Finally, to determine the protein-protein interactions associated with genes, we used the ComPPI database to filter out interactions with non-co-localized subcellular localization, introduced localization scores and interaction scores, and ultimately obtained the proteins that interact with genes (Veres et al., 2015).

### 2.6 Identification of chemical substances interacting with R3HDM1

We utilized the GSCALite database (http://bioinfo.life.hust.edu.cn/web/GSCALite/) to analyze gene expression and drug sensitivity (Liu et al., 2023). GSCALite provides 750 small molecule drugs from GDSC and CTRP, and explores valuable small molecule drugs related to gene expression data. In addition, the National Cancer Institute (NCI) has established the cancer cell line platform, which has been widely used for drug screening based on relevant gene expression. NCI-60 is a collection of 60 human cancer cell lines from nine different cancer types. The NCI-60 expression data comes from CellMiner, and we analyzed the relationship between R3HDM1 expression and drug sensitivity z-scores and calculated Spearman correlation coefficients (Reinhold et al., 2023). Furthermore, we identified the differential expression of genes in different cancer types, and collected 150 most upregulated or downregulated genes as gene-related markers. The CMAP_gene_signatures.RData file contains 1,288 compound-related features, downloaded from the database website (https://www.pmgenomics.ca/bhklab/sites/default/files/downloads) and used for calculating matching scores (Malta et al., 2018). The analysis process followed the methods outlined in previous publications, and the results for 32 cancer types were summarized and graphically presented using the R language (Yang et al., 2022).

### 2.7 Survival and clinical features analysis of R3HDM1 in pan-cancer

Survival data was retrieved from the TCGA database, and the “survival” and “survminer” R packages were used to analyze the correlation between gene expression and prognostic indicators [including overall survival (OS), disease-specific survival (DSS), progression-free interval (PFI), and disease-free interval (DFI)]. We combined two methods, Kaplan-Meier and univariate Cox analysis, to comprehensively determine whether the gene is a risk factor or protective factor, and ultimately created a gene survival map. It is worth noting that when using the Kaplan-Meier method for survival analysis, the best cutoff values for high and low expression queues of R3HDM1 were determined using the R package “survminer,” and the survfit function was used to perform a log rank test to evaluate the significance of high and low expression groups of the gene. In addition, the “forestplot” package was used to visualize the results of Cox analysis of survival data. Furthermore, we used the Wilcoxon rank-sum test and Kruskal–Wallis Rank Sum Test to detect gene expression in different stages and molecular subtypes.

### 2.8 Construction of cell lines expressing R3HDM1 siRNA

H1299 and A549 cells were acquired from Wuhan Sevilla Biotechnology Company, while BEAS-2B cells were obtained from Hunan Prattze Biotechnology Company. The cells were cultured in a CO2 incubator maintained at 37°C with a 5% CO2 atmosphere. Sterile distilled water, sterilized under high-temperature and high-pressure conditions, was regularly replaced in the incubator to maintain appropriate humidity levels. The targeting sequences of R3HDM1 siRNA and the sequence of the negative control siRNA can be found in Table 1. H1299 and A549 cells were seeded into separate culture dishes and transfected when the cell density reached 60%, with mRNA expression levels evaluated after 24 h of standard cultivation.

**TABLE 1:** List of siRNAs.

Primer	Sequence (5′to 3′)
NC	UUC​UCC​GAA​CGU​GUC​ACG​UTT
GAPDH	TCT​TCC​TCT​TGT​GCT​CTT​G
R3HDM1-1	CGC​CAG​AUA​UUU​AGA​GUU​AAU
R3HDM1-2	UGG​GAA​GUC​UGU​CAU​AGU​AAA
R3HDM1-3	AGA​CUU​UCA​GAA​ACG​UUA​UAU
R3HDM1-4	GAG​AGC​CAG​AGA​CCG​AAU​AUU

### 2.9 RT-PCR and RT-qPCR

RNA was extracted utilizing the TRIzol reagent (Invitrogen Life Technologies; Carlsbad, CA, United States), followed by treating RNA samples with the gDNA remover reagent (GenStar) according to the manufacturer’s protocol to eliminate genomic DNA. Subsequently, cDNA synthesis was performed using the RT-Phusion kit (Thermo Fisher Scientific; Waltham, MA, United States). Gene-specific mRNA levels were quantified through standard and quantitative RT-PCR (RT-qPCR) techniques, employing the ΔΔCt method. The primer sequences are available in Table 2.

**TABLE 2:** List of primer.

Primer	Sequence (5′to 3′)
GAPDH-F	GGC​TCT​CCA​GAA​CAT​CAT​C
GAPDH-R	TCT​TCC​TCT​TGT​GCT​CTT​G
R3HDM1-F	GCAGCACAGATTCAGACA
R3HDM1-R	ACC​AGA​AGA​CTC​AGA​ACC​T

### 2.10 Detection of cell migration and proliferation

After transfecting A549 and H1299 cells, they were individually seeded into six-well plates. Once reaching approximately 90% confluency, vertical scratch wounds were created using a 200 μL pipette tip. The scratches were photographed every 12 h, and the migration rate was analyzed using ImageJ software. Suspensions of transfected A549 and H1299 cells were prepared for cell counting purposes. Each well of a 96-well plate was seeded with 1 × 10^4 cells. Subsequently, after cell adhesion, the absorbance at 450 nm was measured every 24 h upon the addition of CCK-8 reagent to calculate the proliferation rate between the negative control group and the treatment group.

## 3 Results

### 3.1 Expression of R3HDM1 in pan-cancer

**FIGURE 1:**
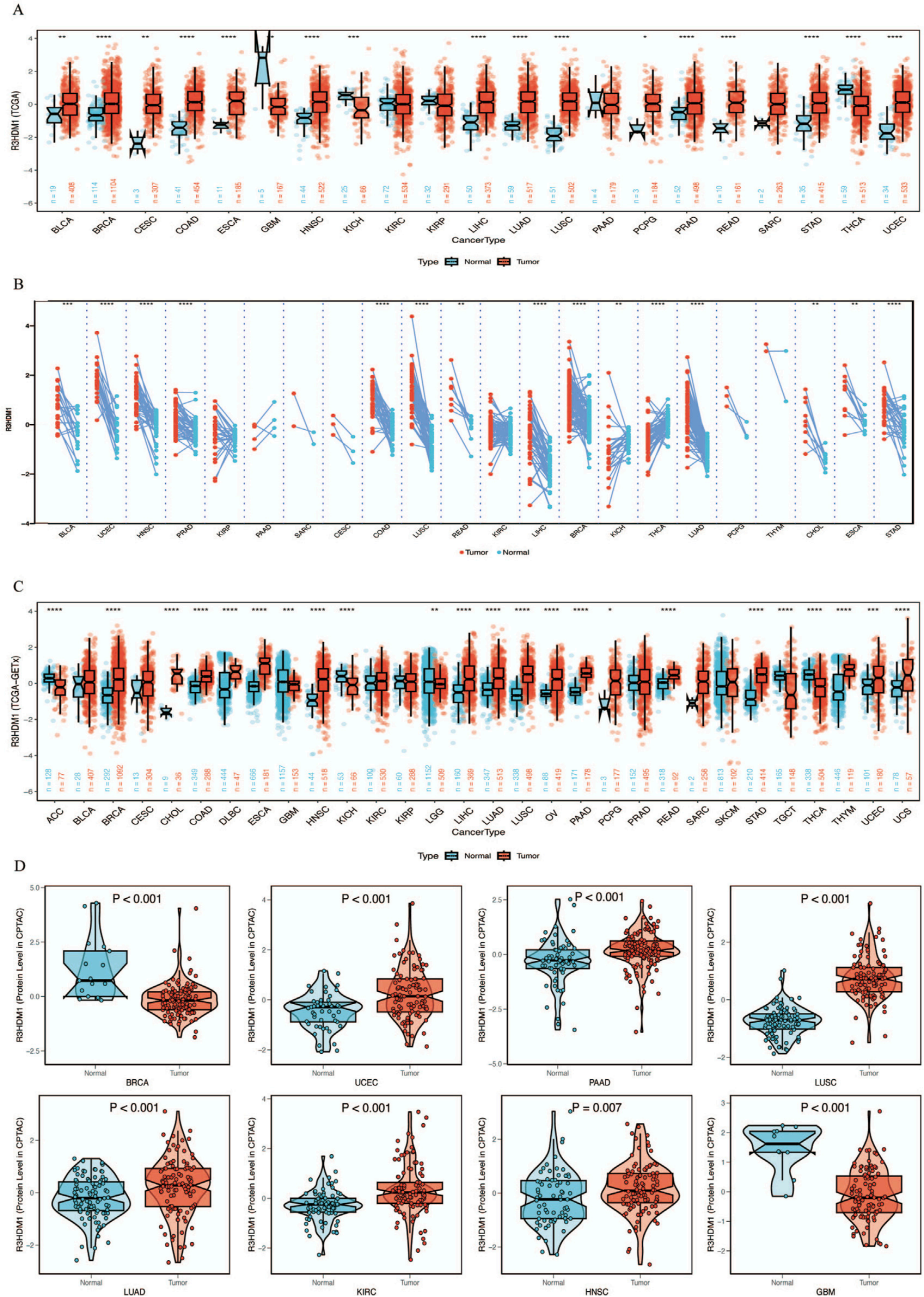
mRNA expression levels of R3HDM1 in human normal and tumor tissues. **(A)** mRNA expression levels of R3HDM1 in normal and cancer tissues using data from the TCGA database. **(B)** mRNA expression levels of R3HDM1 in paired tissues using data from the TCGA database. **(C)** The expression level of R3HDM1 in different tumor tissues and corresponding normal tissues from TCGA and GTEx datasets. **(D)** External validation of protein levels was performed using a CPTAC database. The symbols *, **, and *** indicate *P* < 0.05, *P* < 0.01, *P* < 0.001, respectively.

By combining and mining the resources of TCGA and GTEx databases, we obtained the mRNA expression levels of R3HDM1 from a pan-cancer perspective. Firstly, compared to normal tissues in the TCGA database, we observed significantly higher expression of R3HDM1 in most cancer tissues, including breast cancer (BRCA), Colon adenocarcinoma (COAD), Esophageal carcinoma (ESCA), Head and Neck squamous cell carcinoma (HNSC), Liver hepatocellular carcinoma (LIHC), Lung adenocarcinoma (LUAD), Lung squamous cell carcinoma (LUSC), Pheochromocytoma and Paraganglioma (PCPG), Rectum adenocarcinoma (READ), Stomach adenocarcinoma (STAD), and Uterine Corpus Endometrial Carcinoma (UCEC), among which the differential expression level in lung adenocarcinoma is significantly significant (Figure 1A). Significant differences in expression levels of LUAD were also observed in paired tissues (Figure 1B). Due to the limited number of normal samples in the TCGA data, we combined the GTEx database to further expand the sample size, and obtained consistent results. The significance of the differential expression level in lung adenocarcinoma has not changed (Figure 1C).

**FIGURE 2:**
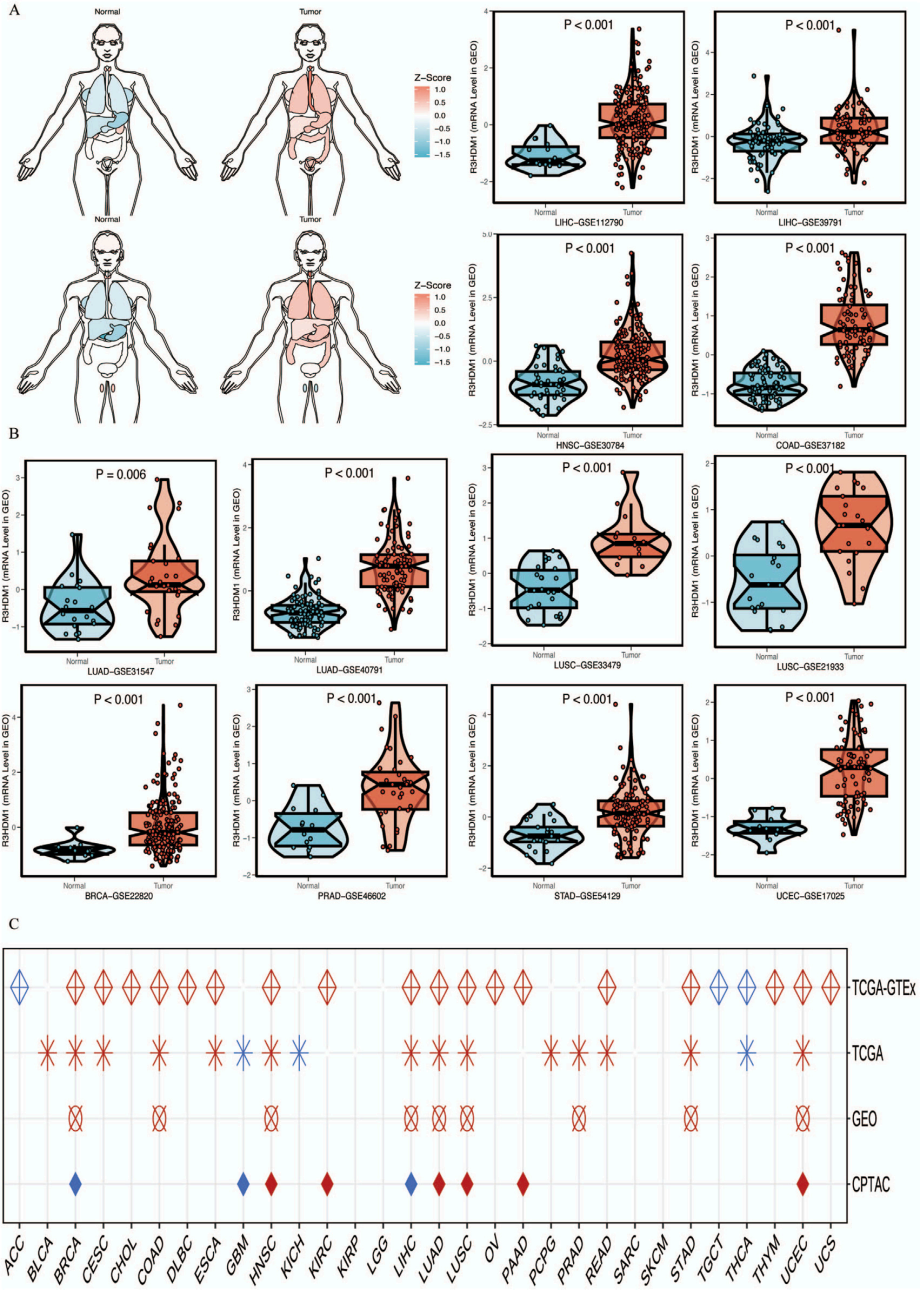
mRNA expression levels of R3HDM1. **(A)** Expression and distribution of R3HDM1 in various organs. **(B)** External validation of mRNA levels was performed using GEO database. **(C)** Logistic regression analysis of TCGA, TCGA-GTEx. Red means OR is greater than 1, blue represents an OR value between 0 and 1. A white circle means that there is no significance, and an empty value means that no relevant data set has been collected in the current database. Tumor tissues are represented by red, while normal tissues are represented by blue.

Next, we visualize the expression distribution pattern of R3HDM1 using organ graphs (Figure 2A). It is evident that, except for the testis, thyroid, brain, and kidney, cancer tissues show significantly higher expression of R3HDM1 compared to normal tissues in other organs, with lung cancer tissues still exhibiting significantly higher expression than normal lung tissue. We found differential expression of R3HDM1 in most types of cancer, and consistent expression patterns across cancer types, with a universal significant upregulation. External validation of mRNA levels was conducted using the GEO database (Figure 2B) and protein levels using the CPTAC database (Figure 1D). Logistic regression analysis based on TCGA, TCGA-GTEx, GEO, and CPTAC datasets fully verified the above results, with good consistency in expression trends across different omics and databases (Figure 2C). The results related to LUAD further confirm the presence of R3HDM1 as a risk factor, warranting further research. In fact, after expanding the sample size of normal tissues by combining the GTEx database, the estimated ROC curve indicates that R3HDM1 demonstrates satisfactory sensitivity and specificity in the diagnosis of 17 cancer types (area under the curve >0.7, Supplementary Figures 1, 2). The reproducibility and consistency of these results have been demonstrated across multiple databases, tumor types, various methods, and omics analyses, suggesting that the dysregulation of R3HDM1 expression may be involved in different cancers and is unlikely to be attributed to technical artifacts, randomness, or biases in sample identification standards within databases.

### 3.2 Genetic variants of R3HDM1 in cancer

**FIGURE 3:**
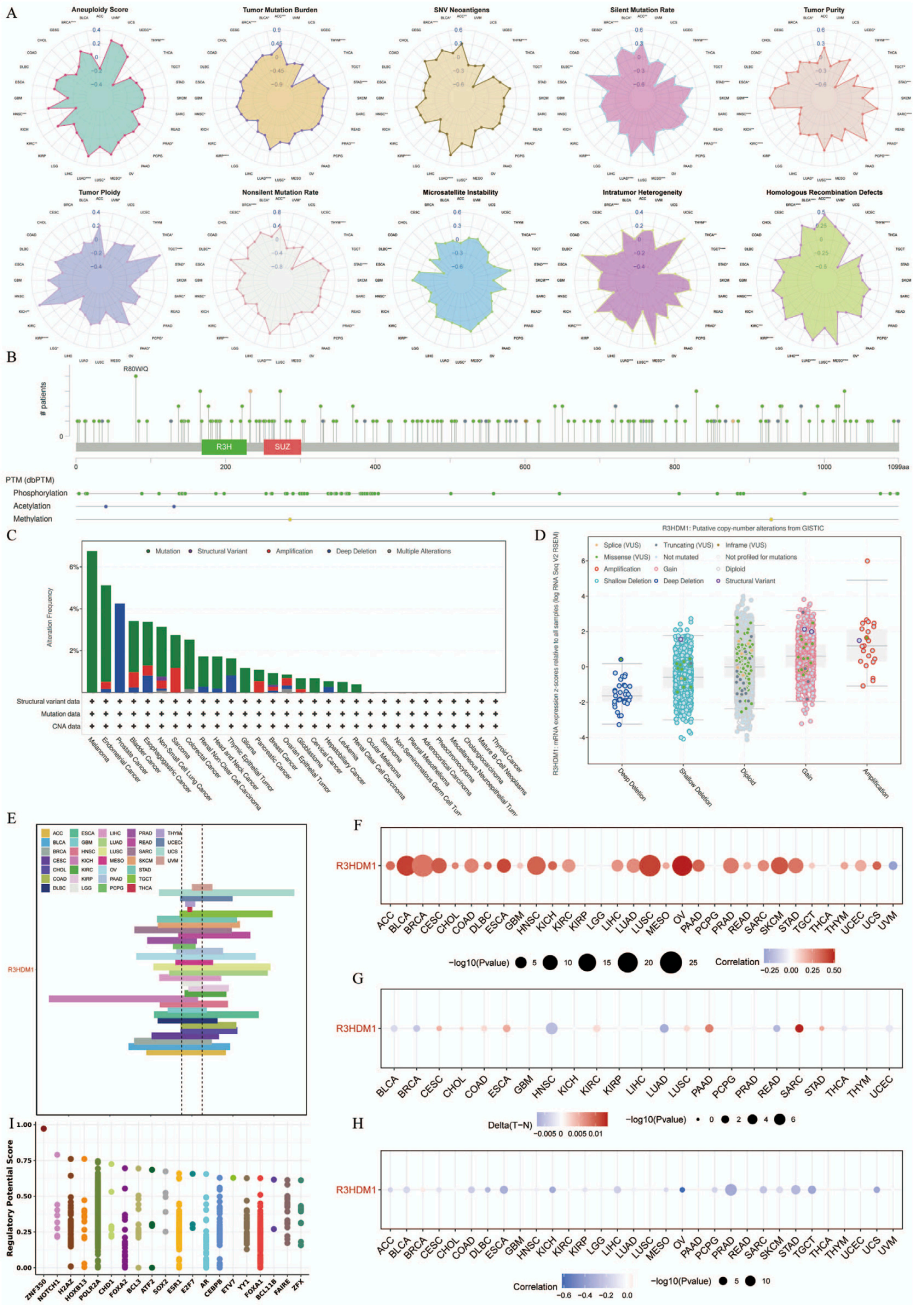
Genetic Alterations of R3HDM1 in cancers. **(A)** Radar map visualization of spearman correlation coefficients of R3HDM1 and 10 genomic features. Statistical significance was reported at *P< 0.05, **P < 0.01, ***P < 0.001, and ****P < 0.0001. **(B)** Sites and number of cases with R3HDM1 genetic alterations in pan-cancer from cBioPortal. **(C)** Frequency of R3HDM1 mutations in different tumor types. **(D)** Relationship between R3HDM1 mRNA expression and genetic alterations. **(E)** Histogram shows the frequency of somatic copy number alterations for R3HDM1 in each cancer type. **(F)** The Spearman’s correlation between somatic copy number alterations and the expression of R3HDM1. **(G)** Heatmap shows the differential methylation of R3HDM1 in cancers; hypermethylated and hypomethylated R3HDM1 are marked in red and blue, respectively (Wilcoxon rank-sum test). **(H)** Spearman’s correlation of R3HDM1 between transcriptional expression and promoter methylation. Red and blue represent positive and negative correlations, respectively. **(I)** Top 20 factors are showed in this plot. Y-axis represents the RP score. X-axis represents different factors. Dots in a x-axis line means same factor.

We obtained genomic data of tumors and normal tissues from the TCGA Pan-Cancer dataset, including genetic variations, somatic copy number alterations (SCNA), mRNA expression, and DNA methylation data. It is worth noting that Figure 3A shows the correlation between R3HDM1 and ten genomic features scores, so we further calculated the relationship between R3HDM1 and different mutation scores, especially the importance of SNV Neoantigens, TMB, and MSI as important biomarkers for immunotherapy. The high SNV Neoantigens score of R3HDM1 expression is positively correlated with Adrenocortical carcinoma (ACC), Bladder Urothelial Carcinoma (BLCA), BRCA, HNSC, Kidney renal papillary cell carcinoma (KIRP), LUSC, LUAD, Prostate adenocarcinoma (PRAD), and Thymoma (THYM). Similarly, the expression of R3HDM1 is positively correlated with high TMB scores in ACC, BLCA, BRCA, HNSC, KIRP, LUSC, LUAD, PRAD, THYM, and STAD. Additionally, the high MSI scores in Lymphoid Neoplasm Diffuse Large B-cell Lymphoma (DLBC), HNSC, Mesothelioma (MESO), KIRP, LUSC, Thyroid carcinoma (THCA), Rectum adenocarcinoma (READ), Skin Cutaneous Melanoma (SKCM), and STAD are positively correlated with R3HDM1 expression. Simultaneously, R3HDM1 expression is significantly positively correlated with the high score of LUAD in eight scoring methods, indicating that R3HDM1 can be used as a predictive marker for the effectiveness of immunotherapy in LUAD. Figure 3B depicts the 2D structure of the R3HDM1 mutation site. Analysis using the cBioPortal database, we found that R3HDM1 exhibits certain genetic alteration frequencies in most cancers, with amplification and mutation being the most common types of genetic alterations (Figure 3C). The mutation frequencies of R3HDM1 are different in different cancers, with the highest mutation frequencies in melanoma, endometrial cancer, prostate cancer, bladder cancer, esophageal gastric cancer, and non-small cell lung cancer. The mutation patterns of R3HDM1 vary in different tumors, predominantly mutations in most tumors, but copy number loss is predominant in prostate cancer and thymic epithelial tumors. At the same time, a significant increase in R3HDM1 expression with the progression of SCNA from copy number loss to amplification (Figure 3D). Clearly, SCNA played a crucial role in regulating gene expression in cancer. We next found SCNA occurs at a high frequency (exceeding 5% of all samples) in most cancer types, with very low frequencies only in THCA and THYM (Figure 3E). Next, we evaluated the impact of SCNA on R3HDM1 expression, and gained the Spearman correlation between R3HDM1 expression and copy number across different cancers in TCGA (Figure 3F). The results indicate a significant positive correlation between R3HDM1 expression and SCNA in most tumors. To further study the genetic alterations of R3HDM1 in pan-cancer, we examined the percentage of SCNA. Then, we observed diverse methylation patterns of R3HDM1 in the pan-cancer dataset (Figure 3G), with lower methylation in tumor tissues compared to normal tissues in HNSC, LUAD, and READ, while higher methylation levels were observed in tumor tissues in Sarcoma (SARC), Pancreatic adenocarcinoma (PAAD), Esophageal carcinoma (ESCA), and COAD. After all, we obtained a negative correlation between the expression of R3HDM1 and DNA methylation (Figure 3H). In addition, we showed that the top three transcription factors with the highest RP scores are ZNF350, NOTCH1, and H2AZ (Wang et al., 2013) (Figure 3I).

### 3.3 The correlation between R3HDM1 and immune microenvirnoment in cancer

**FIGURE 4:**
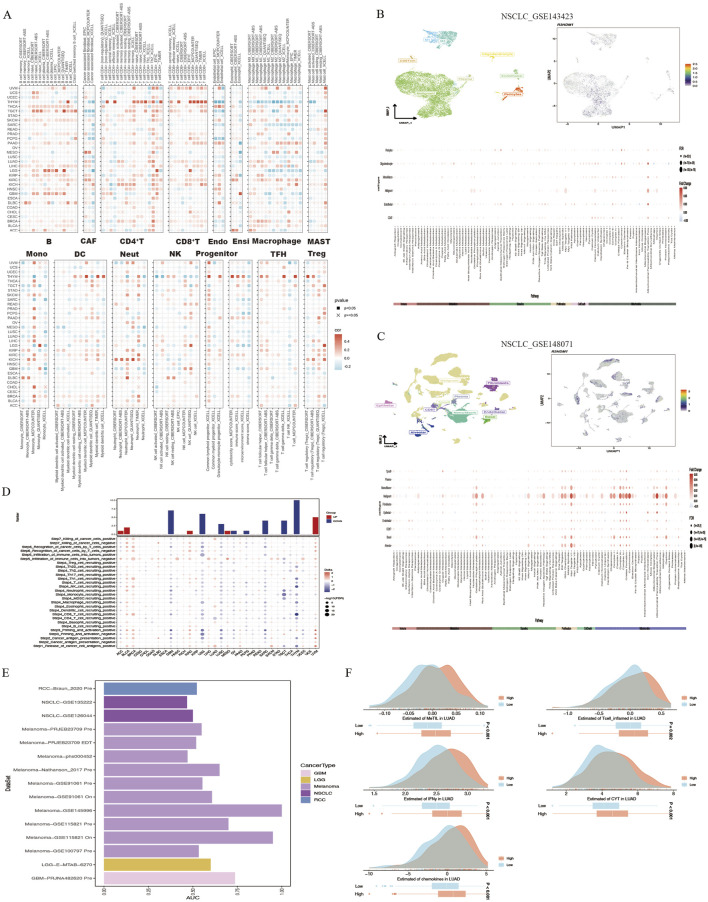
High R3HDM1 expression correlates with immune infiltration genes and immunotherapy in cancer. **(A)** Seven software were used to evaluate the correlation between R3HDM1 expression and cancer immune. **(B)** Correlation between R3HDM1 expression and expression of different cell subsets in the GSE143423 dataset. **(C)** Correlation between R3HDM1 expression and expression of different cell subsets in the GSE148071 dataset. **(D)** The difference of TIP scores between high and low expression groups of R3HDM1 was calculated. **(E)** Prediction of immune therapy response and ROC-AUC value in unresponsive patients with pan cancer R3HDM1 expression. **(F)** Differences in five scoring methods in the high and low expression groups of R3HDM1.

The interaction between tumor cells and the tumor microenvironment is crucial for tumor development, progression, metastasis, and treatment response. Analysis of the correlation between R3HDM1 expression and immune infiltration levels in TCGA tumor profiles using seven different algorithms revealed a significant positive association of R3HDM1 expression with B cells, CD4^+^T cells, CD8^+^T cells, and Treg cells in nearly all cancer types. This suggests that R3HDM1 plays a role in immune rejection or immune silence, highlighting its critical involvement in the interactions between the immune system and tumors, especially in immune evasion (Figure 4A). To further study the cell types expressing R3HDM1 in tumor tissues, we analyzed single-cell expression levels of R3HDM1 (Supplementary Figure 7), finding widespread expression in various immune and malignant cells. For instance, UMAP plots and heat maps demonstrate a significant positive correlation of R3HDM1 expression with CD8^+^T cells and proliferative T cells (T-prolif) in non-small cell lung cancer (NSCLC_GSE143423 and NSCLC_GSE148071), indicating its significant role in cellular proliferation, cell death, and mitochondrial energy metabolism pathways (Figures 4B, C). Additionally, R3HDM1 demonstrates a reverse expression pattern between T-prolif and exhausted CD8^+^T cells in multiple cancer tissues, suggesting its potential regulatory role in T cell function (Supplementary Figure 7).

Given the regulatory role of R3HDM1 expression levels in the immune response and immune cell infiltration across various human cancers from our previous analyses, we proceeded to investigate the predictive role of R3HDM1 in cancer immunotherapy. To assess this, we downloaded data from the TIP database and evaluated the activity scores of the cancer immunity cycle using the GSVA algorithm, discovering a positive correlation between high R3HDM1 expression and the release of cancer cell antigens (step 1), recruitment of Th1 cells (step 4), and T cell recognition of cancer cells (step 6). However, in most cancer types, a negative correlation was observed between high R3HDM1 levels and the recruitment of CD4^+^T cells (step 4), recruitment of CD8^+^T cells (step 4), and immune cell infiltration into tumors (step 5), further indicating the immunosuppressive role of R3HDM1 in the TME (Figure 4D).

Subsequently, from a pan-cancer perspective on the response to immunotherapy, we visualized the predictive capability of R3HDM1 for predicting responders and non-responders to pan-cancer immunotherapy (Figure 4E). We found varied predictive performance of R3HDM1 across different datasets, with the best performance observed for Melanoma and good performance for RCC, NSCLC, LGG, and GBM. Finally, we used MeTIL scores, CYT scores, IFN-γ scores, Tcell_inflamed scores, and Chemokines scores to predict the response of LUAD patients receiving immune checkpoint inhibitor (ICB) therapy in the LUAD patient dataset. We observed that LUAD patients with high R3HDM1 expression exhibited a significantly favorable response to ICB therapy, indicating the effective predictive ability of R3HDM1 for the response rate of LUAD patients to immune therapy (Figure 4F).

### 3.4 The relationship between R3HDM1 and pathways

**FIGURE 5:**
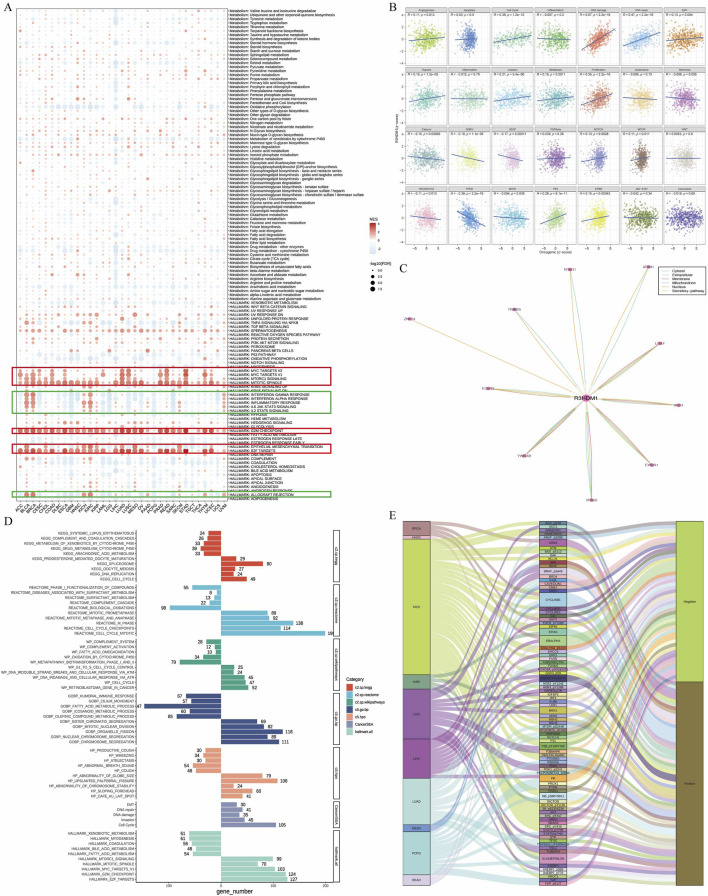
Association between gene and pathways in cancer. **(A)** Enrichment differences of R3HDM1 in 50 HALLMARK and 85 metabolism gene sets. NES is the normalized enrichment score in the GSEA algorithm. **(B)** R3HDM1 expression was highly correlated with 28 malignant features of the LUAD. **(C)** Obtain the interaction information of R3HDM1, in which the color of the line corresponds to the data source, and the length of the line corresponds to the interaction score. **(D)** The enrichment analysis results for R3HDM1 in LUAD are presented in the figure. The bar on the right illustrates the enrichment of highly expressed genes, whereas the bar on the left depicts the opposite scenario. **(E)** Sankey’s picture clearly shows the proteins associated with R3HDM1 in the TCPA database.

To further understand the impact of R3HDM1 on the prognosis of cancer patients, especially LUAD, we conducted Gene Set Enrichment Analysis (GSEA). Firstly, we examined the differentially expressed genes between high and low R3HDM1 patients in each cancer type. The result shows that in the high R3HDM1 expression group of almost all cancer types, cell proliferation-related signaling pathways (including MYC, mTORC1, spindle, G2M, and E2F pathways) are significantly enriched (Figure 5A). This finding corroborates previous findings of sustained high expression of R3HDM1 in malignant cells, indicating its crucial role in tumor cell proliferation. Furthermore, in BLCA, KICH, KIRC, KIRP, LUAD, SKCM, and MESO patients, high expression of R3HDM1 is positively correlated with epithelial-to-mesenchymal transition (EMT). This correlation may explain why patients with high levels of R3HDM1 in SKCM cancer type are prone to distant metastasis. Interestingly, the enrichment of the EMT pathway is not significant in patients with high levels of R3HDM1 in THCA and TGCT, which is consistent with the analysis of staging status in these two types of cancer, where the expression levels of R3HDM1 in stages I and II are significantly higher than in stages III and IV (Supplementary Figure 5). In addition, the heatmap also highlights differential enrichment of immune-related pathways, such as interferon (IFN)-γ, IFN-α, inflammation, IL-6, IL-12, complement, and allograft rejection pathways (Figure 5A). These pathways are negatively enriched in most cancer patients with high R3HDM1 expression levels, including CESC, DLBC, ESCA, GBM, HNSC, LAML, LGG, LUSC, MESO, OV, PCPG, SARC, and UCS. These findings suggest that R3HDM1 may be involved in inhibiting the anti-tumor immune response in these cancers. It is worth noting that, for LUAD patients, high levels of R3HDM1 are also associated with immune response, IL-6, IL-12, and complement pathways, albeit to a moderate degree. In summary, these results strongly indicate that the elevated levels of R3HDM1 are closely associated with increased proliferation, EMT, and immune suppression in human cancers. Additionally, we analyzed the relationship between the scores of 14 cancer-related pathways in LUAD and R3HDM1 (Figure 5B). Obviously, the scores related to the cell cycle are higher and positively correlated than other scores, which further confirms the sustained expression of R3HDM1 in malignant cells, indicating its involvement in tumor cell proliferation. Moreover, using comPPI, helped us identify the related genes that may interact with R3HDM1 and represented the interaction sites with different colored lines, suggesting that R3HDM1 plays a wide range of roles through the networks (Figure 5C). At the same time, we found in the high R3HDM1 expression group of LUAD, pathways related to the cell cycle, cell proliferation (including MYC, mTORC1, spindle, G2M, and E2F pathways), and epithelial-mesenchymal transition (EMT) were significantly enriched (Figure 5D). This finding provides additional evidence that R3HDM1 is consistently highly expressed in malignant LUAD cells, and is strongly associated with tumor progression, proliferation, and metastasis. Meanwhile, we investigated proteins in the TCPA database with a correlation of over 0.35 with R3HDM1 and visualized them. We found a positive correlation between the expression levels of R3HDM1 and various functional proteins in LUAD (Figure 5E). Specifically, the expression of R3HDM1 in LUAD is positively correlated with the functional proteins TFRC, MTOR_pS2448, and X4EBP1, which are known to be involved in key cellular processes such as metabolism, apoptosis, signaling, cell cycle regulation, and proliferation.

### 3.5 The association between R3HDM1 and chemotherapy

**FIGURE 6:**
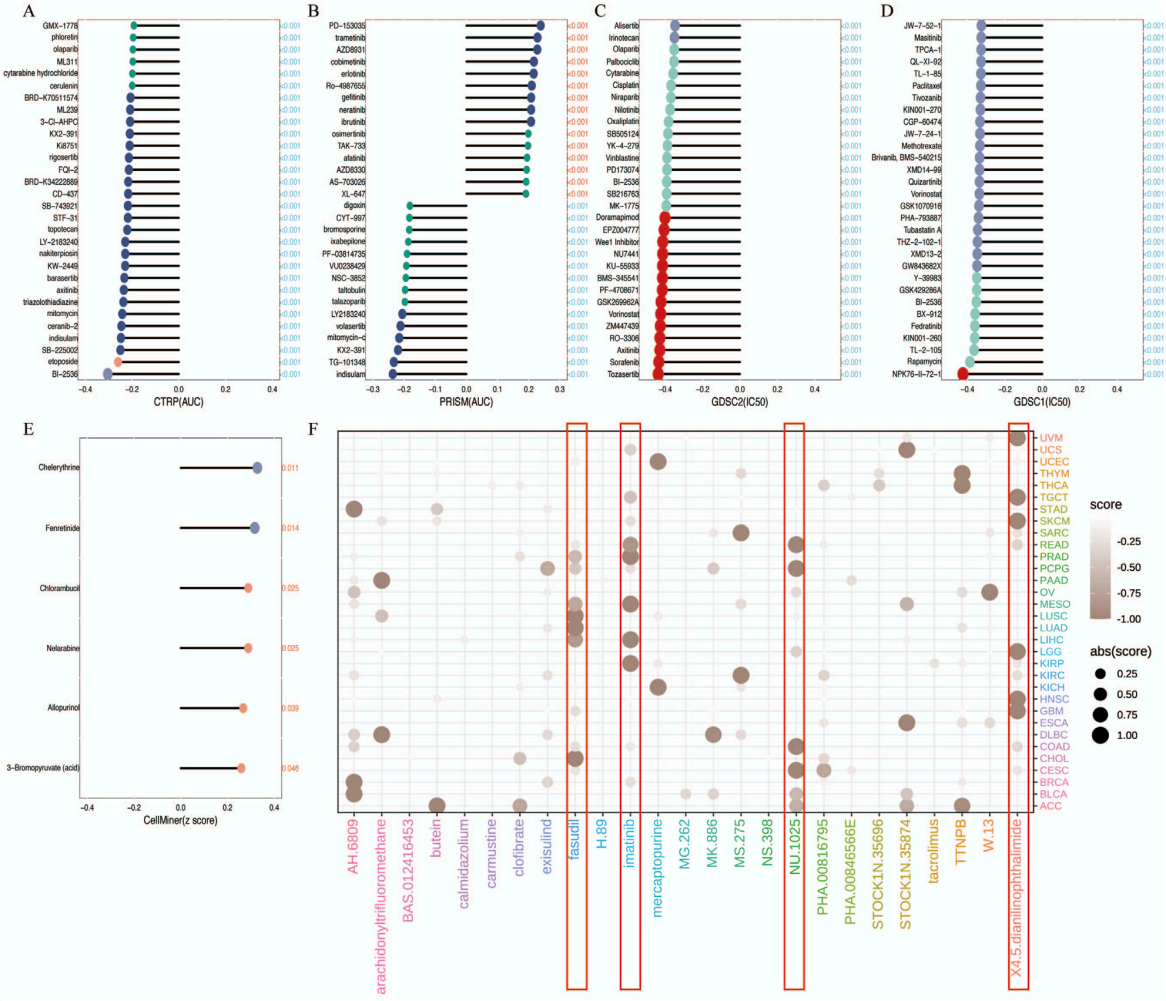
Analysis of Treatment and Drug Resistance. **(A–E)** Drug sensitivity analysis based on R3HDM1 expression using the four different databases Cellminer, CTRP, GDSC, and PRISM. *P* < 0.05 was considered statistically significant. GSCA provides a bubble plot to summarize the correlations between inputted genes and drugs. Only when a gene associated with at least one drug will be obtained. Also, only when a drug associated with at least one gene will be obtained. Blue bubbles represent negative correlations, red bubbles represent positive correlations, the deeper of color, the higher of the correlation. Bubble size is positively correlated with the FDR significance. The black outline border indicates FDR ≤ 0.05. Note that GSCA only draws plot for the top 30 ranked drugs. The drugs are ranked by the integrated level of correlation coefficient and FDR of searched genes. In detail, firstly, drug-gene pairs with an absolute correlation coefficient > 0.1 and FDR< 0.05 were remained. The −log_10_FDR and the absolute value of the correlation coefficient were then multiplied to calculate a score for each gene-drug pair. Finally, the sum of the scores was calculated for each drug. The top 30 drugs with the highest scores are presented in the figure. The genes in the plot are ranked in the same way. **(F)** Prediction of potential compounds targeting R3HDM1. Visualized the top5 candidate compounds, based on connectivity map analysis of 32 cancer.

In the chemotherapeutic analysis, we investigated the potential correlation between drug sensitivity and R3HDM1 expression using four different databases (Figures 6A–E) It illustrate a substantial negative correlation between the sensitivity AUC and IC50 of numerous drugs across the CTRP, PRISM, and GDSC databases. This suggests that elevated R3HDM1 expression renders cells highly responsive to a wide array of drugs. Conversely, in the Cellminer database, R3HDM1 showed a significant positive correlation with the z scores of all drugs. Clearly, R3HDM1 emerges as a potential chemosensitive gene. To explore potential therapeutic strategies that can inhibit the tumor-promoting effects mediated by R3HDM1, we conducted CMap analysis. We constructed a set of R3HDM1-related gene signature, comprising the top 150 upregulated and 150 downregulated genes, determined by comparing patients with high and low gene expression within each cancer type. Utilizing the optimal feature matching method XSum (eXtreme Sum), we compared the gene-related features with CMap gene features, resulting in similarity scores for 1,288 compounds. From the result, fasudil, imatinib, NU1025, and 4,5-Dianilinophthalimide exhibited significantly lower scores across most cancer types, suggesting their potential to inhibit R3HDM1-mediated oncogenic effects (Figure 6F). Surprisingly, previous studies have demonstrated the anti-tumor effects of fasudil, imatinib, and 4,5-Dianilinophthalimide. These findings substantiate the validity of our predictive outcomes, although further research is warranted to elucidate the underlying mechanisms.

### 3.6 Clinical relevance of R3HDM1

**FIGURE 7:**
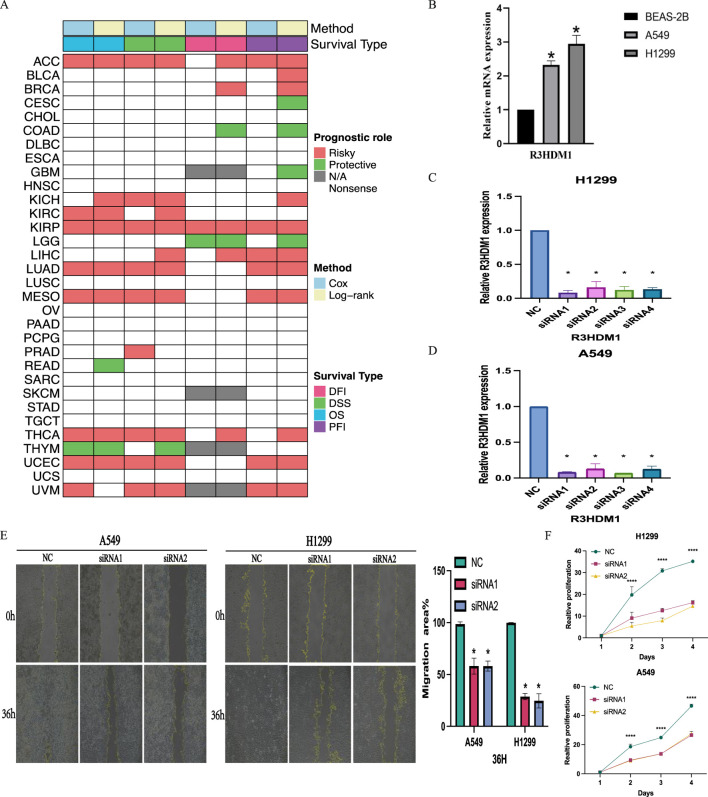
Clinical Relevance of R3HDM1. **(A)** Heatmap showing the correlation between R3HDM1 expression levels and four curated survival outcomes. Red boxes represent a risk factor, green boxes represent a protective factor, white boxes represent the analyses are not significant, and gray boxes represent the data are not available. **(B)** Relative mRNA expression in cell lines. **(C)** Determination of knockdown efficiency of R3HDM1 in H1299. *P < 0.05; n = 3 **(D)** Determination of knockdown efficiency of R3HDM1 in A549. *P < 0.05; n = 3 **(E)** The effect of knocking down R3HDM1 on the migration of lung adenocarcinoma cells. *P < 0.05; n = 3 **(F)** The effect of knocking down R3HDM1 on the proliferation of lung adenocarcinoma cells. *P < 0.05; n = 3.

The study explored the clinical relevance of R3HDM1 in cancer by analyzing its role in cancer survival. Survival analysis across various cancer types revealed a consistent association of R3HDM1 with multiple survival periods, primarily as a risk factor for various cancers, notably in LUAD. While R3HDM1 typically acts as a risk factor at different survival stages in most tumors, exceptions exist where it serves as a protective factor; for instance, higher R3HDM1 expression is linked to better survival rates in LGG, THYM, and similar conditions. These results underscore the diverse role of R3HDM1 in different cancers, highlighting the need for further investigation into its functional significance in cancer survival. Clustering results (Figure 7A) underscored R3HDM1’s prognostic value for 11 tumor types, including ACC, BRCA, KICH, KIRC, KIRP, LUAD, MESO, THCA, UCEC, LIHC, and UVM. Moreover, forest plots and Kaplan-Meier curves (Supplementary Figures 3, 4) presented the Cox survival rate analysis and log-rank test outcomes across various cancers. We conducted further investigation into the correlation between R3HDM1 expression and tumor staging, revealing its association with multiple tumor stages (Supplementary Figure 5), implying a link between R3HDM1 and the progression of these tumors. Notably, R3HDM1 displayed variations across various molecular subtypes in different cancers (Supplementary Figure 6). For instance, it exhibited the lowest expression in the LumA subtype of BRCA, higher expression in the Basal subtype, and significantly higher expression in Triple-Negative Breast Cancer (TNBC). In LUSC, its expression was lowest in the Basal subtype and highest in the Primitive subtype. The expression across different subtypes in LUAD varied significantly. These findings underscore the precision molecular stratification, therapeutic implications, and prognostic value of R3HDM1 in diverse cancers. At last, the normalized RT-qPCR results show substantial variations in R3HDM1 expression levels between normal lung glandular epithelial cells and lung adenocarcinoma cells (Figure 7B). We assessed the efficiency of R3HDM1 knockdown in A549 and H1299 cells post transfection with small interfering RNA (siRNA) using RT-qPCR. The results indicated significant transfection efficiencies for all four siRNA sequences, with siRNA1 and siRNA3 showing the most effective knockdown efficiency (*P* < 0.05) (Figures 7C, D). Subsequently, siRNA1 and siRNA3 were selected for further cellular experiments. Following this, we conducted cell scratch and CCK8 cell viability assays to examine the impact of R3HDM1 knockdown on the migration and proliferation abilities of A549 and H1299 cells. The results demonstrated a significant decrease in migration ability in cell lines with R3HDM1 knockdown compared to the negative control group (*P* < 0.05) (Figure 7E). Furthermore, after 24 h of R3HDM1 knockdown expression, the proliferation capabilities in the treatment group exhibited a significant decrease compared to the negative control group (*P* < 0.0001). The experimental findings are in line with the aforementioned prognosis analysis (Figure 7A) and GSEA results (Figure 5A). In conclusion, our study highlights the significant role of R3HDM1 in the development and progression of lung adenocarcinoma.

## 4 Discussion

R3HDM1 is an uncharacterized RNA-binding protein containing an R3H domain. Previous studies have suggested R3HDM1 as a potential candidate oncogene (Tuupanen et al., 2014). Additionally, ARPP21 (also known as R3HDM3), belonging to the same protein family as R3HDM1, has been implicated as a potential key oncogene in tumors (Fukushi et al., 2021; He et al., 2021). Research has shown that ARPP21 can interact with mutant p53, leading to the inhibition of miR-128-2 targeting E2F5 protein, thereby enhancing the impact of mutant p53 on tumorigenesis, progression, and chemoresistance (He et al., 2021; Donzelli et al., 2012). However, the relationship between R3HDM1 of the same protein family and cancer remains unexplored.

This study, for the first time, comprehensively elucidated the dysregulated expression of R3HDM1 in pan-cancer using diverse tools, suggesting its potential as a diagnostic biomarker across multiple cancers. Additionally, it systematically analyzed the relationship between R3HDM1 expression and clinical outcomes in pan-cancer, identified potential drugs and small molecules targeting R3HDM1 from various dimensions, delineated cell populations expressing R3HDM1 at high resolution through extensive single-cell datasets, characterized R3HDM1-mediated pathways and metabolic disruptions, identified transcription factors regulating its expression through chip-seq analysis, associated R3HDM1 expression with various molecular subtypes, highlighting the potential for stratified precision therapy and exploring its underlying mechanisms. Our findings indicated upregulated R3HDM1 expression in most tumors, including LUAD (Figure 2C). Moreover, significantly increased protein expression of R3HDM1 was noted in LUAD (Figure 2C). The study also revealed correlations between R3HDM1 expression and pathological staging in LUAD, ESCA, LIHC, PAAD, SKCM, and THCA (Supplementary Figure 5). Overall, these results strongly suggest that R3HDM1 may exhibit specific or contrasting functions across different tumor types, particularly in LUAD.

To further elucidate the functionality of R3HDM1 in various types of tumors, this study investigated the correlation between R3HDM1 expression and patient prognosis. It was observed that the expression levels of R3HDM1 were significantly correlated with the prognosis of 19 types of cancer when analyzing the prognostic predictive abilities using Overall Survival (OS), Disease-Specific Survival (DSS), Disease-Free Interval (DFI), and Progression-Free Interval (PFI) (Supplementary Figures 3A–D). High expression of R3HDM1 was identified as a risk factor for the occurrence and progression of tumors, particularly in six types of cancers (ACC, LUAD, MESO, UCEC, THCA, and KIRP). Additionally, the association between R3HDM1 expression levels and clinical characteristics in different types of cancers was evaluated. Consistent with the survival analysis results, R3HDM1 expression was correlated with staging in eight tumors, including LUAD, implying its involvement in the progression of these tumors (Supplementary Figure 5). The higher expression of R3HDM1 in the metastatic group in melanoma further indicates its role in tumor metastasis. Interestingly, differences in R3HDM1 expression were found across various molecular subtypes of 17 cancers, including LUAD, suggesting its involvement in tumor progression and its potential for precise molecular stratification therapy and predictive value (Supplementary Figure 6). These findings were further supported by the results of Gene Set Enrichment Analysis (GSEA) (Figure 5A). In summary, these results confirm that the expression levels of R3HDM1 can serve as a reliable biomarker for predicting the prognosis of patients with tumors, including LUAD.

Ten scoring methods correspond to reliable biomarkers for the prognosis of various cancers, serving as predictive factors for the effectiveness of many tumor immunotherapies, particularly TMB, MSI, and SNV neoantigens (Darragh et al., 2018; Chen et al., 1992; Capietto et al., 2022). Tumors with high TMB scores, high MSI scores, and high SNV neoantigens scores demonstrate better responses to immunotherapy (Reck et al., 2019; Repáraz et al., 2022; Chang et al., 2018; Li K. et al., 2020). Interestingly, the expression of R3HDM1 in KIRP, HNSC, and LUSC positively correlates with SNV neoantigens, TMB, and MSI (Figure 3A). Therefore, we speculate that high R3HDM1 expression in KIRP, HNSC, and LUSC may lead to greater survival benefits post-immunotherapy, suggesting R3HDM1 as a potential novel drug target for anticancer immunotherapy.

The tumor microenvironment (TME) comprises diverse cells and serves as the locale for tumor cell, invasive immune cell, and stromal cell accumulation (Wu and Dai, 2017). In recent years, advancements in immunotherapy have unveiled the significant association between immune cell infiltration and tumor initiation, progression, and treatment (Wu and Dai, 2017; Sadeghi Rad et al., 2021). Consequently, exploring the link between R3HDM1 and immunity is imperative. Our findings unveil the multifaceted involvement of R3HDM1 in immune microenvironment remodeling across distinct tumors. Remarkably, we observed a significant positive correlation between R3HDM1 expression and B cells, CD4^+^T cells, CD8^+^T cells, and Treg cells in nearly all cancer types, hinting at R3HDM1’s partial participation in immune evasion or immune tolerance, pivotal in immune-tumor interplay, notably immune escape. Analyses of immune regulatory function revealed associations between R3HDM1 expression and diverse tumor immune scores, indicating a potential role for R3HDM1 in modulating the tumor microenvironment. Subsequent scrutiny unveiled a positive correlation between R3HDM1 expression and Th2 and TFH cell infiltration, while displaying inverse relationships with central and effector memory CD4^+^T and NKT cells (Figure 4A). Th2 cells, recognized for their tumor-promoting and immunosuppressive functions, were notably highlighted (Liu et al., 2019; Frafjord et al., 2021). Conversely, CD4^+^T cells and NKT cells, pivotal in tumor eradication, showcased reduced infiltration in high R3HDM1 tumors, fostering an immunosuppressive tumor backdrop (Liu et al., 2021; Borst et al., 2018). Furthermore, we assessed the anti-cancer immune status across the seven stages of the cancer immune cycle (Figure 4D). Notably, in most cancer types, heightened R3HDM1 levels exhibited a negative correlation with immune cell infiltration within tumors and antigen-presenting molecule expression, contrasting with a positive correlation observed with various immune negative regulatory molecules. These observations align with the results from Gene Set Enrichment Analysis (GSEA), endorsing R3HDM1’s role in mediating immune suppression and impacting the efficacy of immunotherapy within the tumor microenvironment. Analyzing an extensive corpus of single-cell data revealed that R3HDM1 exhibits high expression levels in malignant cells and T-related cells (Supplementary Figure 7). These findings underscore the close association between R3HDM1 expression and immune cell infiltration in tumors, influencing tumor onset, progression, and patient prognosis, thus offering novel targets for immunosuppressant development.

## 5 Conclusion

Multi omics studies have elucidated the dysregulation of R3HDM1 expression in pan cancer, which is a potential diagnostic marker for pan cancer and has been confirmed in lung adenocarcinoma cell lines. Elaborated on the pathways and metabolic disorders mediated by R3HDM1, and identified the transcription factors regulating its expression using chip-seq. By combining a large number of single-cell datasets, the cells expressing R3HDM1 were identified at high resolution. Clarified the possibility of targeting R3HDM1 from multiple dimensions, drugs and small molecules. The system describes the relationship between R3HDM1 and clinical outcomes in pan cancer. Linking R3HDM1 expression with multiple molecular subtypes demonstrates the potential for stratified precision therapy.

## Figures and Tables

**Table:** 

**ACC**	Adrenocortical carcinoma
**AUC**	Area under the curve
**BLCA**	Bladder urothelial carcinoma
**BRCA**	Breast invasive carcinoma
**CESC**	Cervical squamous cell carcinoma and endocervical adenocarcinoma
**CHOL**	Cholangiocarcinoma
**CCLE**	Cancer cell line encyclopedia
**COAD**	Colon adenocarcinoma
**COAD/READ**	Colon adenocarcinoma/Rectum adenocarcinoma esophageal carcinoma
**DSS**	Disease-specific survival
**DFI**	Disease-free interval
**DLBC**	Lymphoid neoplasm diffuse large B-cell lymphoma
**ESCA**	Esophageal carcinoma
**FAP**	Fibroblast activation protein-α
**GTEx**	Genotype Tissue-Expression
**GSEA**	Gene set enrichment analysis
**GSVA**	Gene set variation analysis
**GBM**	Glioblastoma multiforme
**GBMLGG**	Glioma
**HNSC**	Head and Neck squamous cell carcinoma
**ICIs**	Immune checkpoint inhibitors
**KICH**	Kidney chromophobe
**KM**	Kaplan-Meier
**KIRC**	Kidney renal clear cell carcinoma
**KIRP**	Kidney renal papillary cell carcinoma
**LAML**	Acute myeloid leukemia
**LGG**	Brain lower grade glioma
**LIHC**	Liver hepatocellular carcinoma
**LUAD**	Lung adenocarcinoma
**LUSC**	Lung squamous cell carcinoma
**MESO**	Mesothelioma
**NCI**	National Cancer Institute
**OS**	Overall survival
**OV**	Ovarian serous cystadenocarcinoma
**PFI**	Progress-free interval
**PPI**	Protein-protein interaction
**PAAD**	Pancreatic adenocarcinoma
**PCPG**	Pheochromocytoma and paraganglioma
**PRAD**	Prostate adenocarcinoma
**ROC**	Receiver operating characteristic curve
**SARC**	Sarcoma
**SKCM**	Skin cutaneous melanoma
**STAD**	Stomach adenocarcinoma
**STES**	Stomach and esophageal carcinoma
**TCGA**	The Cancer Genome Atlas
**TIICs**	Tumor-infiltrating immune cells
**TME**	Tumor microenvironment
**TGCT**	Testicular Germ Cell Tumors
**THCA**	Thyroid carcinoma
**THYM**	Thymoma
**UCEC**	Uterine corpus endometrial carcinoma
**UCS**	Uterine carcinosarcoma
**UVM**	Uveal melanoma

## Data Availability

Data for the TCGA pan-cancer cohort were obtained from the Firehose database (http://gdac.broadinstitute.org), comprising both tumor and normal samples. MiRNA, TCPA, mutation data, molecular subtypes, and clinical information were downloaded from the UCSC Xena database (https://xenabrowser.net/datapages/). External validation data for mRNA levels were sourced from the GEO database (https://www.ncbi.nlm.nih.gov/geo/), while protein-level mass spectrometry data were retrieved from the CPTAC database (https://proteomics.cancer.gov/programs/cptac). Pan-cancer immune infiltration results, assessed using various algorithms, were obtained from the TIMER2.0 database (http://timer.cistrome.org/). It is important to highlight that these public databases are accessible to all users at no cost. This study strictly adheres to the data extraction policies of each database and therefore does not necessitate ethical review and approval from an ethics committee.
