# Supplementary material for: Comprehensive pan-cancer analysis and experiments revealed R3HDM1 as a novel predictive biomarker for prognosis and immune therapy response
Source: Front Genet. 2024 Sep 23;15:1404348. doi: 10.3389/fgene.2024.1404348 (PMC11456529; doi:10.3389/fgene.2024.1404348)
Supplement: Supplementary file 1 [file Table1.DOCX]

Supplementary Material

# Supplementary Data

# Supplementary Figures and Tables

For more information on Supplementary Material and for details on the different file types accepted, please see [here](https://www.frontiersin.org/guidelines/author-guidelines#supplementary-material).

## Supplementary Figures
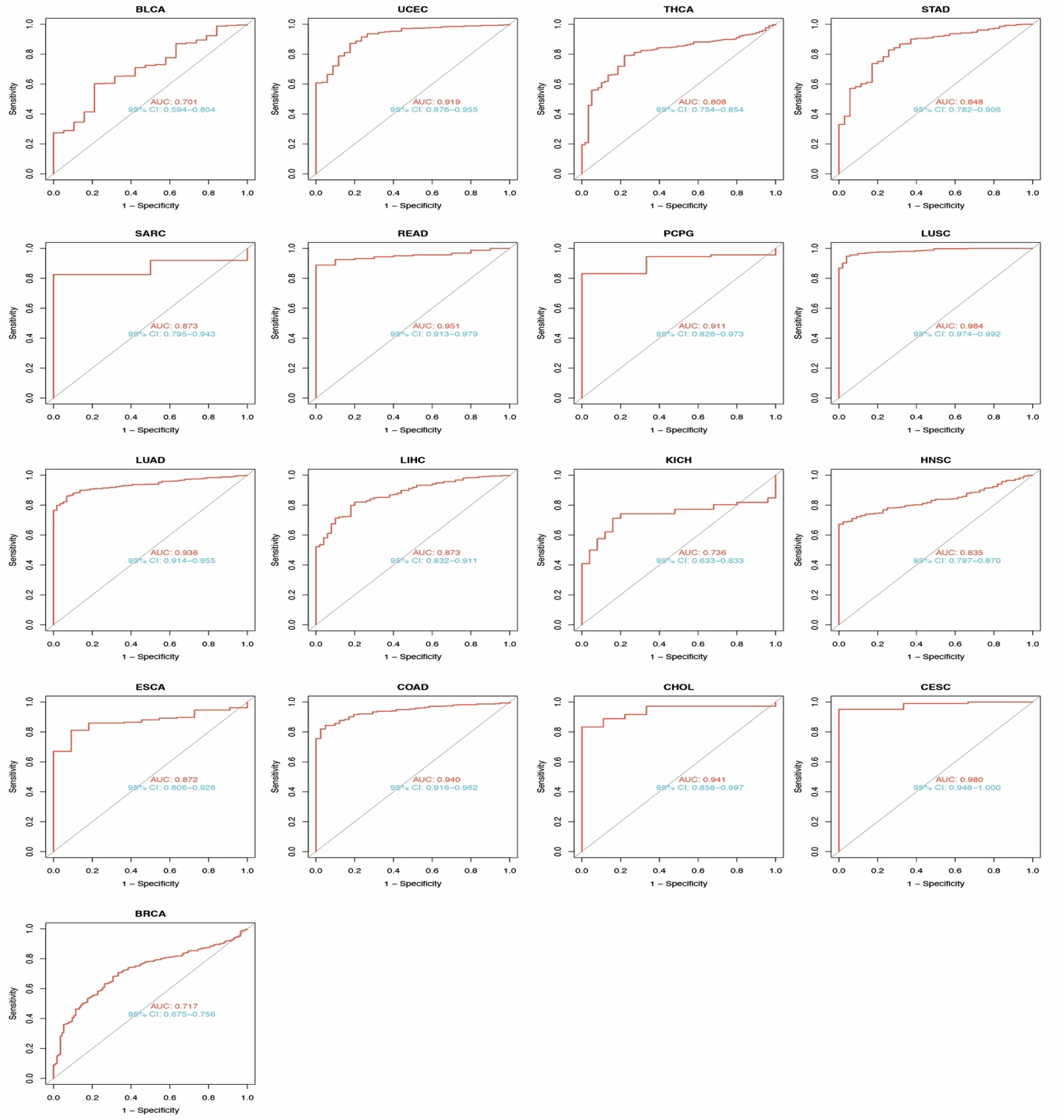


**Supplementary Figure 1.** Diagnostic value of R3HDM1 to distinguish tumor tissues from normal tissues in TCGA. The AUC value was more than 0.7.

**
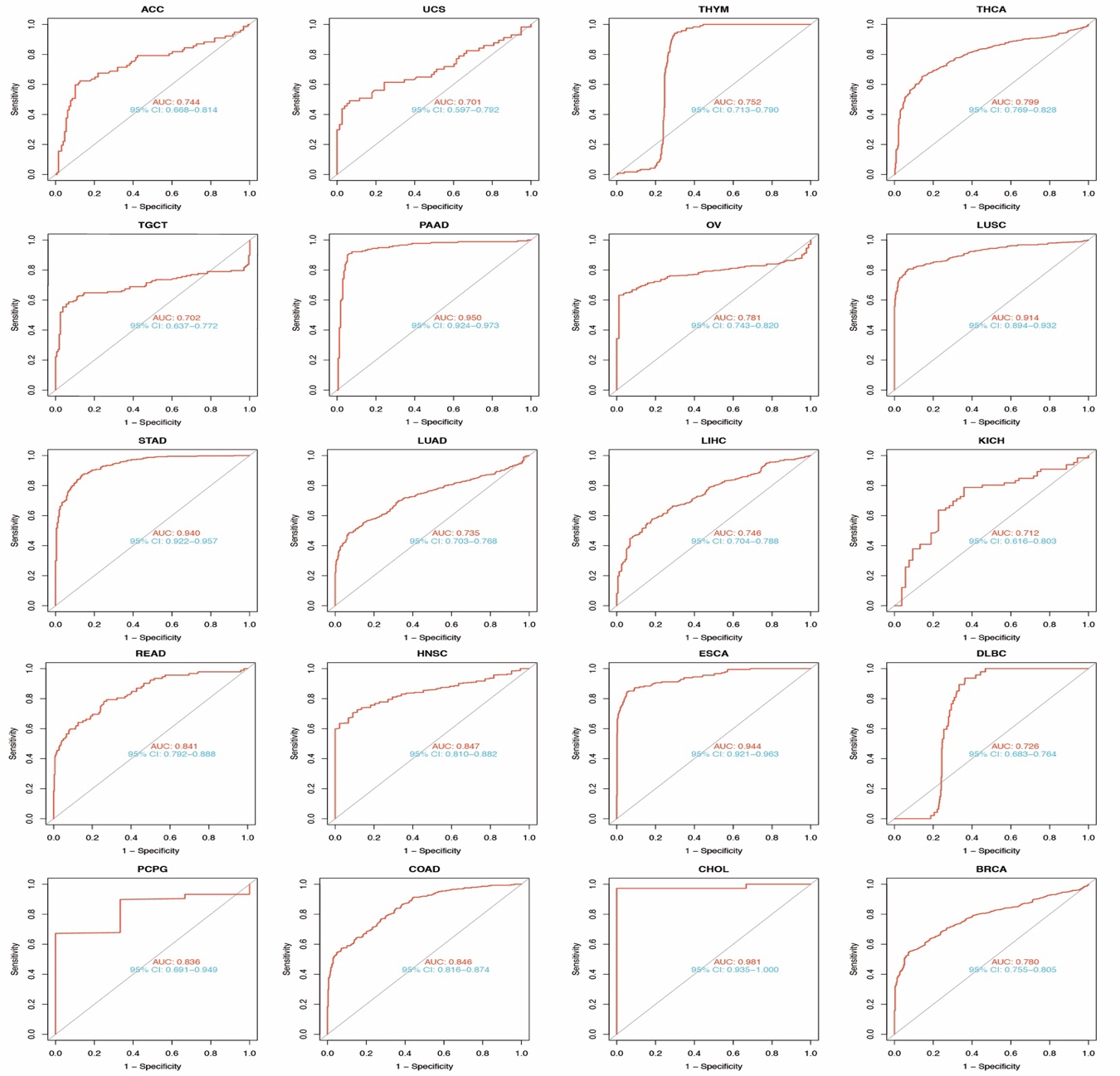
**

**Supplementary Figure 2.** Diagnostic value of R3HDM1 to distinguish tumor tissues from normal tissues in TCGA-GTEx. The AUC value was more than 0.7.


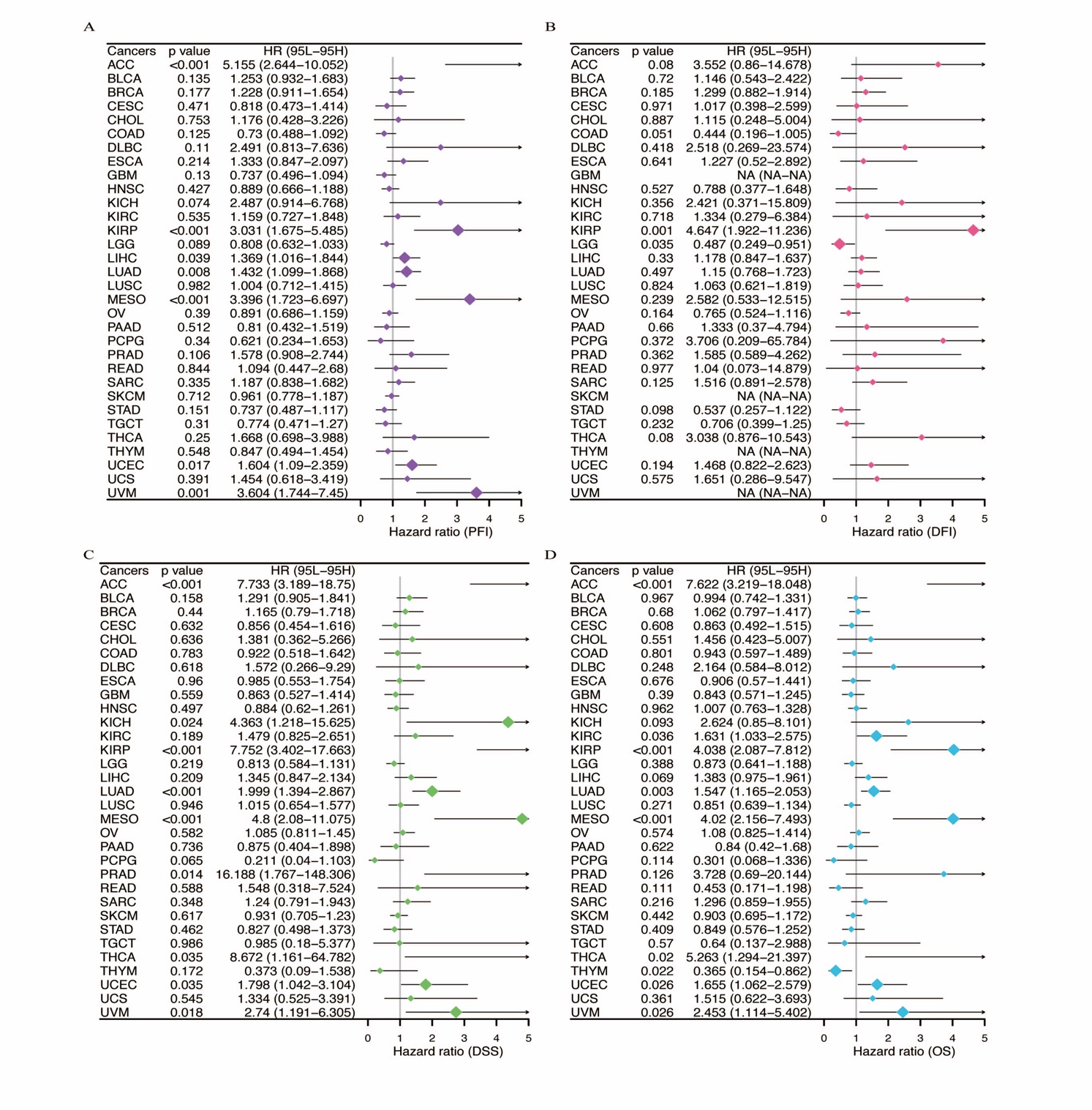


**Supplementary Figure 3.** The forest plot exhibited the prognostic role of R3HDM1 in cancers by univariate Cox regression method.


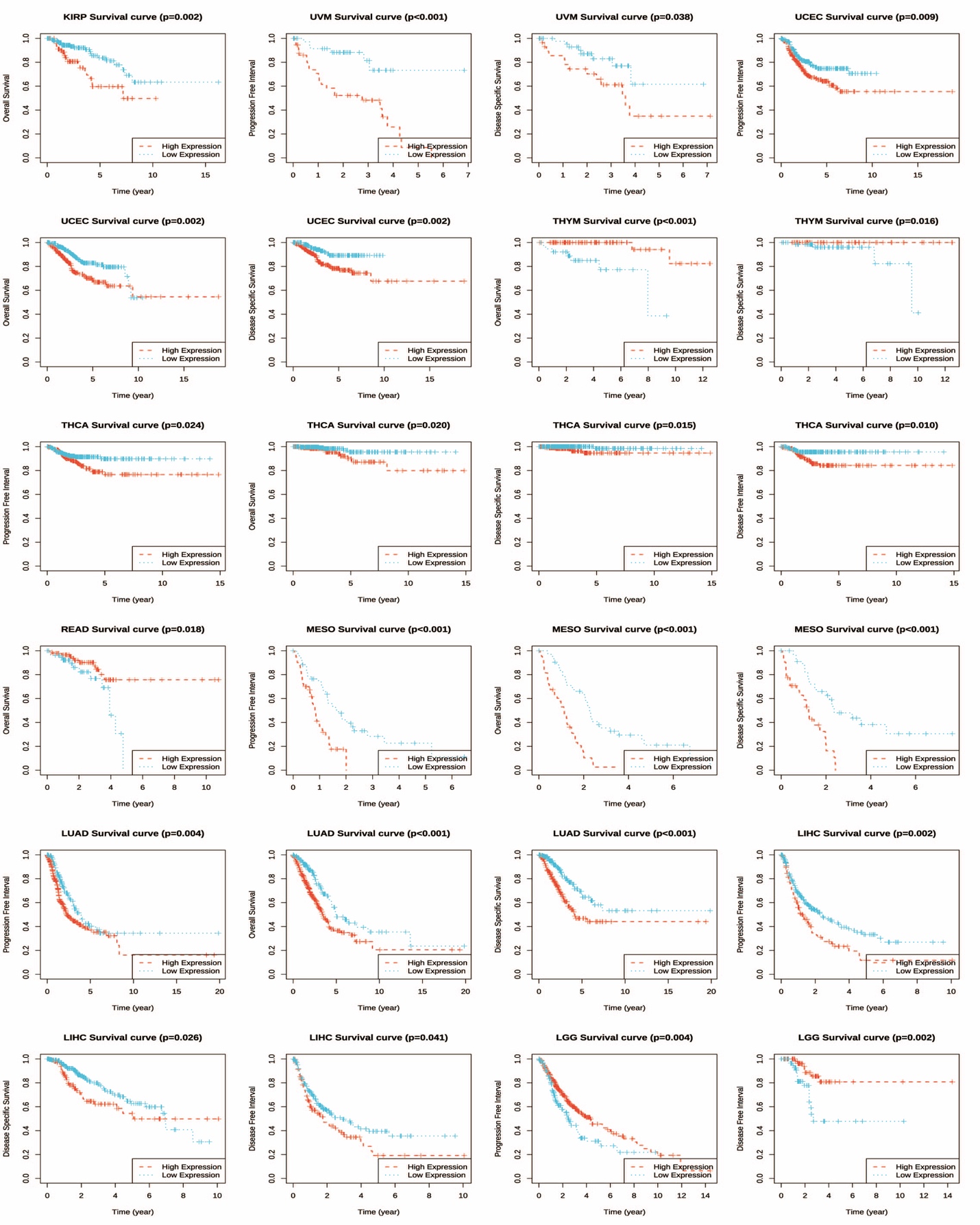


**Supplementary Figure 4.** Kaplan-Meier survival analysis and log-rank testing were performed using "survival" and "survminer" packages.


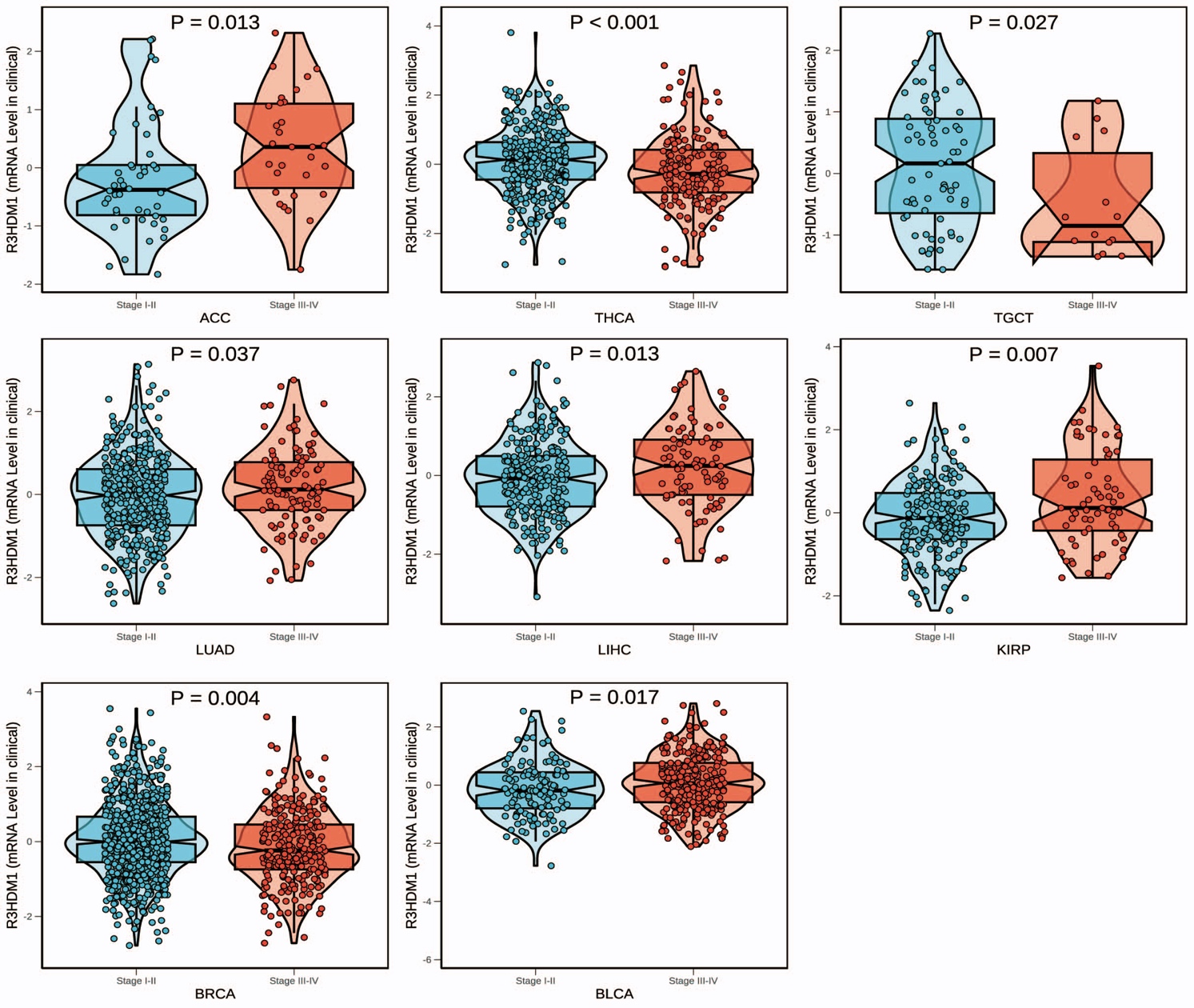


**Supplementary Figure 5.** Wilcoxon rank-sum test examined the differences in R3HDM1 expression in Stages.


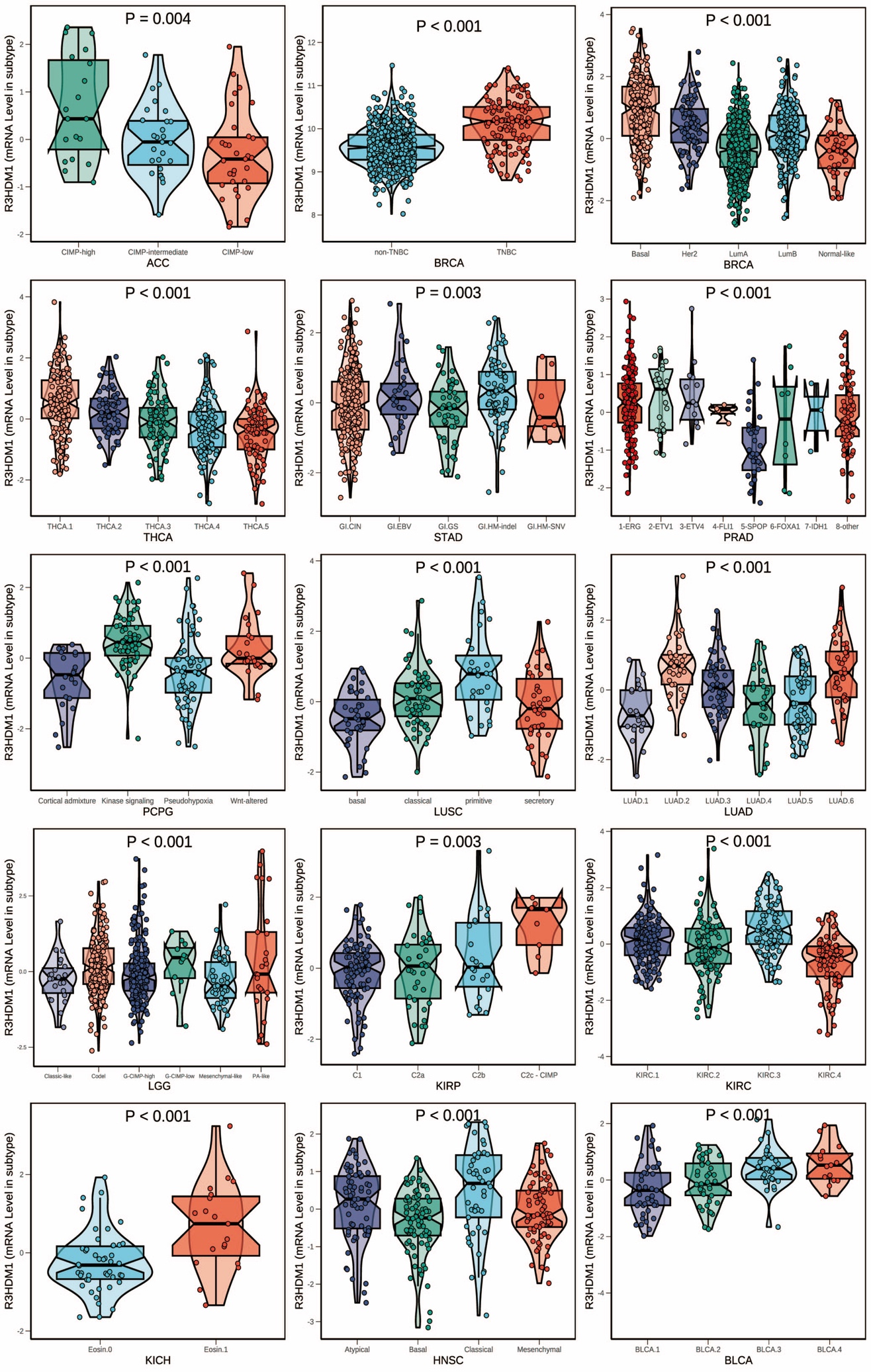


**Supplementary Figure 6.** Kruskal-Wallis examined the differences in R3HDM1 expression in molecular subtypes.

**Supplementary Figure 7.** Cell sources of R3HDM1 in pan-cancer at the single-cell level. The heatmap showed expression distributions of R3HDM1 in different cells among types of cancer.
